# Immune regulation by oral tolerance induces alternate activation of macrophages and reduces markers of plaque destabilization in Apob^tm2Sgy^/Ldlr^tm1Her/J^ mice

**DOI:** 10.1038/s41598-017-04183-w

**Published:** 2017-06-21

**Authors:** Lakshmi Narasimha Thota, Thiruvelselvan Ponnusamy, Sheena Philip, Xinjie Lu, Lakshmi Mundkur

**Affiliations:** 1 Research Scholar, Manipal University, (Madhav Nagar, Manipal) at Molecular Immunology Unit Thrombosis Research Institute, Bangalore, India; 2 Molecular Immunology unit, Thrombosis Research Institute, Bangalore, India; 3 Molecular Immunology unit, Thrombosis Research Institute, London, UK

## Abstract

Atherosclerosis is the leading cause for cardiovascular mortality. We determined the effect of multi-antigenic construct expressing three peptides AHC (ApoB100, HSP60 and outer membrane protein of chlamydia pneumonia) in stabilizing advanced atherosclerosis in Apob^tm2Sgy^/Ldlr^tm1Her/J^ mice. Atherosclerosis was induced by feeding high fat diet (HFD) to mice for 10 weeks, followed by five oral dosing with purified AHC or ovalbumin on alternate days and continued on HFD for another 10 weeks. Tolerance was associated with significantly higher numbers of regulatory T cells both in aortic sinus and spleen with higher mRNA expression of CTLA4 (3 fold), Foxp3 (1.4 folds) and TGF-β (1.62) in aorta. Tregs cells were found to induce alternate activation of macrophages to M2 phenotype, with a reduction in plaque inflammation. AHC treatment showed evidence of plaque stabilization as observed by reduction in plaque necrosis in aortic sinus (35.8%) and in brachiocephalic artery (26%), with reduced expression of Tissue factor and MMP9. Macrophage apoptosis was reduced and collagen content was enhanced by treatment. Our results suggest that tolerance to atherogenic peptides increases regulatory T cells which activate M2 macrophages, prevent T cell proliferation and reduce plaque destabilization and inflammatory markers thus providing evidences for plaque stabilization in mice with advanced atherosclerosis.

## Introduction

Atherosclerosis, the leading cause for cardiovascular diseases is a chronic inflammatory disease with systemic autoimmune etiology^1–3^. Progressive growth of atherosclerotic plaque obstructs the blood flow in the arteries either by constricting the lumen or by formation of thrombus, resulting in a clinical disease manifested as coronary artery disease, stroke or the peripheral arterial diseases^4^. The disease pathogenesis involves retention of lipoproteins inducing a recruitment of immune- inflammatory cells, foam cell formation, apoptosis and necrosis^5–7^. Plaques may remain asymptomatic in a subclinical disease, they may get obstructive as in stable angina or rupture causing acute coronary syndrome (ACS)^8^, which is the most overwhelming cause of mortality worldwide^9,10^.

Several research groups have shown that immune modulation by restoring tolerance to self antigens is a promising therapeutic model for atherosclerosis^11–18^. Oral administration of antigen induces immune tolerance and provides a potential for treating autoimmune and inflammatory diseases^19–21^. Although studies on immune modulation have been successful in reducing the plaque burden in animal models, its effect on advanced atherosclerosis has not been studied in great detail^22–24^. Recent advances in the understanding of molecular basis of plaque progression has helped us to define the markers related to plaque instability^25^. The pathophysiology of vulnerable plaque or thin-cap fibro-atheroma (TCFA) includes disturbances in cellular processes such as growth/differentiation, apoptosis, fibrinogenesis, synthesis of extracellular matrix (ECM) and collagen interface^26,27^. Lower levels of collagen in the intimal layer, defective efferocytosis, accumulation of inflammation in the plaque, macrophage death, necrosis and degradation of extracellular matrix increase the chances of a plaque to rupture^28^.

Earlier studies from our lab has shown that a multi antigenic construct expressing peptide epitopes from apolipoprotein (ApoB), Heat shock protein (HSP60) and Chlamydia pneumonia outer membrane protein (AHC) induces a tolerogenic immune response and reduces the plaque development in mice as well as rabbits^18,29,30^. In the present study, we wanted to understand the curative effect of oral tolerance to AHC protein on stabilizing the makers related to plaque destabilization in the double knock out (Apob^tm25gy^LDLr^tm1Her^) mice model.

## Results

### AHC mediated protection in mice with established disease is associated with regulatory immune response

**Figure 1:**
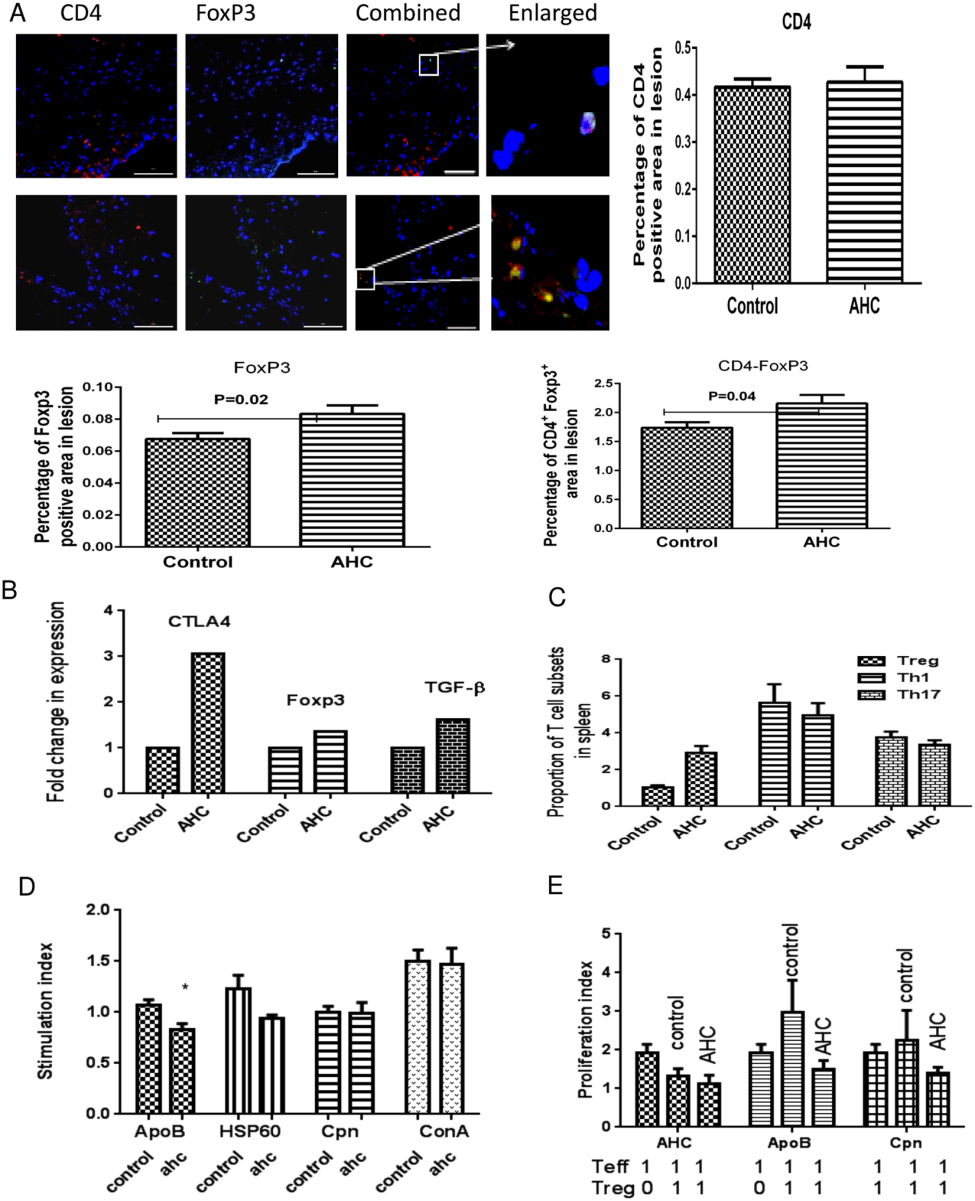
Oral administration of AHC in established disease could induce the tolerance. (**A**) Representative photomicrograph showing immunofluorescence staining of aortic sinus sections with CD4 and Foxp3 and co-localised for CD4^+^FoxP3^+^. Percentage of CD4, FoxP3 and CD4^+^FoxP3^+^ positive area from not less than 18 sections for each group (n = 6). The scale bar represents 200 µm. (**B**) mRNA levels of CTLA4, Foxp3, TGF-β, Arginase1 and Arginase2 in the ascending aorta were quantified using RT-PCR analysis and normalized with GAPDH. Fold changes in expression of the AHC immunized mice relative to control mice are shown, n = 6 per group. (**C**) Flow cytometry analysis of splenic lymphocytes from AHC immunized in hyperlipidemic conditioned mice group and control mice at the end of the experiment. The graph represents percentage of CD25^+^ Foxp3^+^ cells within the CD4 population in spleen, and expression of Th1 as seen by IFNγ and Th17 as seen by IL-17 within the CD4 population, n = 6 per group. (**D**) Proliferation of spleen cells in treated mice as proliferation index was represented as the percentage BrDU reduction in culture stimulated with ApoB, HSp60, Cpn or Concanavalin A (10 ug/ml) relative to control group. *p < 0.05. (**E**) Splenic effector cells were generated from Apob^−^/LdlR^−^ mice immunized subcutaneously with the peptides. Addition of purified Tregcells from oral tolerant mice and control mice to effector cells. Proliferation of effector cells in treated mice as proliferation index was represented as the percentage CFSE reduction in culture stimulated with ApoB, HSp60 or Cpn relative to control group. *p < 0.05.

We have earlier shown that oral tolerance increases the number of regulatory T cells in the aorta as well as spleen of animals when the treatment is given before disease initiation. To understand if the mechanism is active in mice with established atherosclerotic lesions we examined the presence of regulatory T cells (CD4^+^FOXP3 positive cells) in the aortic sinus by immunohistochemistry. The percentage of regulatory cells in the aortic sinus was significantly (p = 0.04) higher in the treated group (2.16 ± 0.15%) when compared to control group (1.74 ± 0.09%) (Fig. 1A). In corroboration with these results oral immune treatment by AHC resulted in 3 fold increases in relative mRNA expressions of CTLA4, 1.4 folds for Foxp3 and 1.62 folds for TGF-β suggesting the dominance of Treg cells in aorta of treated animals (Fig. 1B). Any Immune based therapy to disease could influence the changes in T cell sub population of peripheral lymphoid system. Therefore we examined the changes in Th1, Th17 and Treg cell populations in the spleen. AHC treatment did not shown significant changes in the percentage of Th1 and Th17 cells as seen by IFN-γ positive CD4^+^ cells and IL-17 positive CD4^+^ in the spleen. In contrast, CD4^+^ CD25^+^ Foxp3^+^ cells were significantly higher in number in treated group compared to control (2.94 ± 0.35% vs 1.04 ± 0.11%, p = 0.02) (Fig. 1C). We observed a significant (p = 0.007) reduction in proliferation of splenocytes to the ApoB peptide in treated group and non significant reduction to HSP60 and comparable proliferation to Cpn peptide and concanavalin-A (conA) (Fig. 1D). The regulatory cells purified from AHC treated spleen could suppress the proliferation of AHC specific effector T cells in the presence of all three individual peptides (APOB, HSP60 and Cpn) but this suppression was not statistically significant (Fig. 1E).

### Effect on disease progression upon oral administration of AHC in established disease

**Figure 2:**
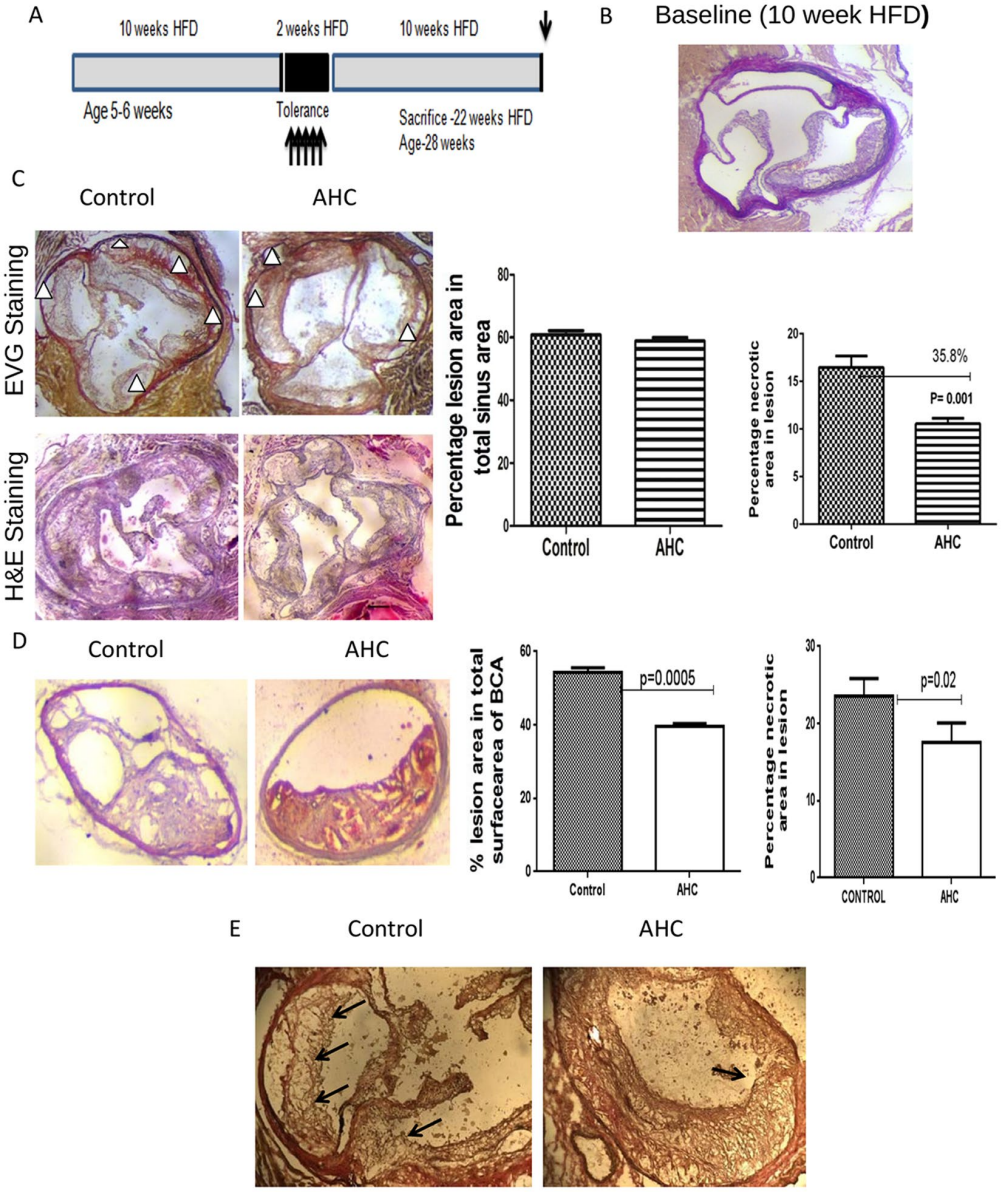
Oral administration of AHC in established disease could maintain the Plaque stability. (**A**) Experimental design. Groups of 6 mice (5–6 weeks, 3M + 3F) were given a diet rich in cholesterol (HFD) for 10 weeks to induce atherosclerosis. Orally dosed with 1 ug of purified recombinant multi antigenic molecule (AHC) for 5 days on alternate days after 10 weeks of HFD. HFD was continued for another 10 weeks. (**B**) Representative photomicrographs of baseline aortic sinus (10 week HFD) aortic sinus plaque area stained with Elastica van Gieson (EVG) stain. Lesion area was 24.5% in total area. (**C**) Representative photomicrographs of aortic sinus plaque area stained with Elastica van Gieson (EVG) stain and hematoxylin and eosin (H&E) (left panels). The hearts were sectioned at the end of the study. The percentages of plaque area in total aortic sinus and necrotic core area were measured and quantified (right panel)from not less than 36 sections from each group. Triangles point to the acellular necrotic core area: p < 0.001. The scale bar represents 200 µm. (**D**) Representative photomicrographs of aortic sinus plaque area stained with hematoxylin and eosin (H&E). (**E**) Representative photomicrographs of 10X zoomed aortic sinus plaque stained with Elastica van Gieson (EVG). Arrows show the thinning and breaks in the cap of plaque.

To understand if the relative increase in Treg cells can also protect the animals against progression of atherosclerosis we then examined the quantity and quality of plaque in the aortic sinus. The experimental design for this study is given in Fig. 2A. The base line group of high fat diet fed mice showed 24.5% plaque deposition before the start of treatment (Fig. 2B). Oral administration of AHC molecule was found to significantly reduce (P = 0.001) plaque necrosis by 35.8% (10.5% ± 0.55 *vs* 16.48% ± 1.17) as quantified by total acellular area in hematoxylin and eosin (H&E) stained aortic sinus in comparison with control group, although the total lesion area was comparable in both control (60.8 ± 2.5%) and treated (57.46 ± 2.56%) animals (Fig. 2C). The brachiocephalic artery of treated animals showed a significant decrease in lesion area by 28% (39.5 ± 0.78% vs 54.4 ± 1.17%, p = 0.0005) as well as necrosis by 26% (17.5 ± 2.53% vs 23.5 ± 2.23%, p = 0.02) in comparison to control group (Fig. 2D). Further the morphometric analysis on aortic sinus also shown less number of breaks in the cap of atheroma by AHC oral treatment compared to control (Fig. 2E).

### Effect of multi-antigenic AHC treatment on decreased plaque instability markers

**Figure 3:**
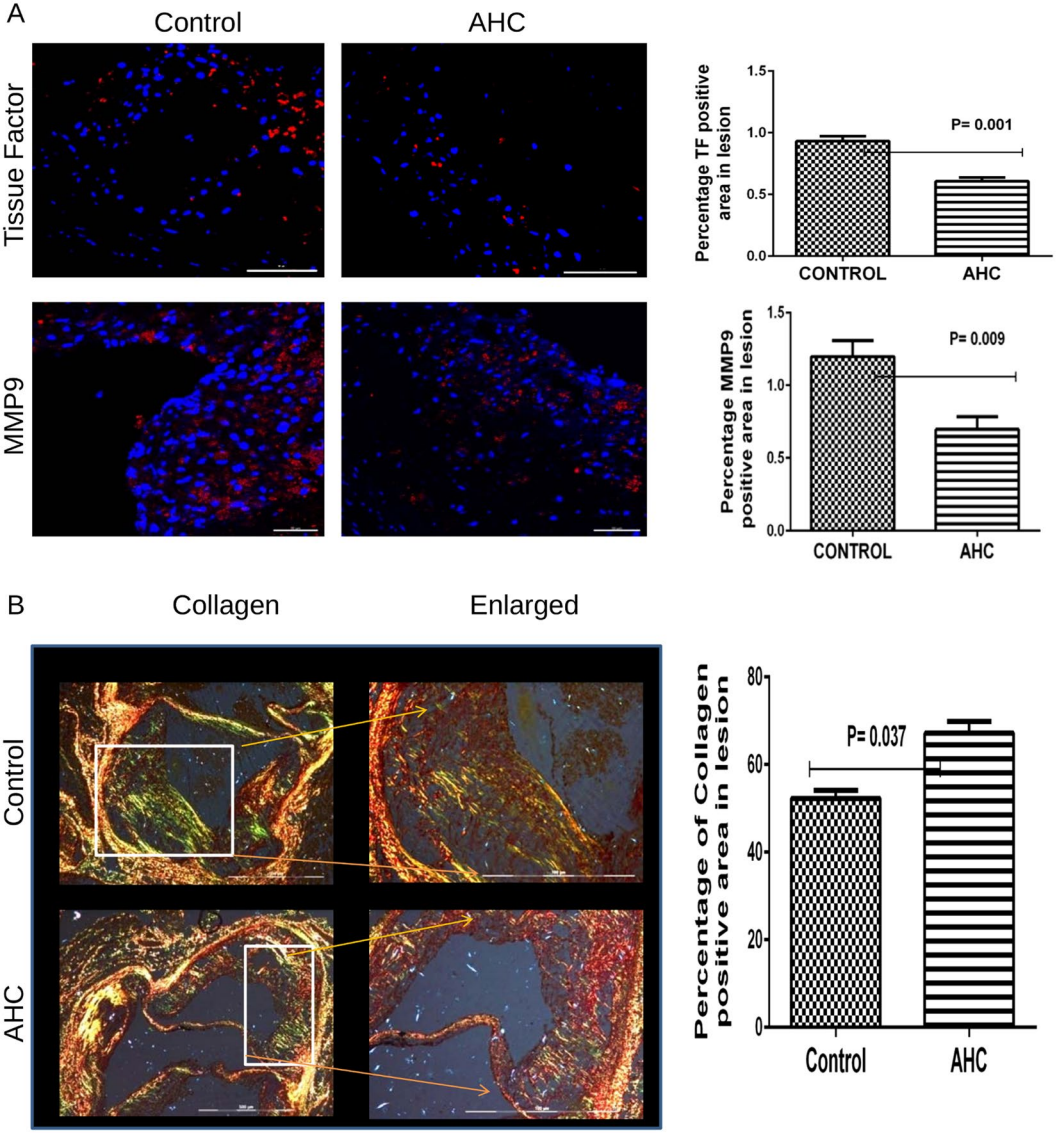
Oral administration of AHC reduces the expression of Plaque rupture markers inturn increases collagen. (**A**) Representative photomicrograph showing immunofluorescence staining of aortic sinus sections with Tissue Factor (TF) and MMP9 (left panel). Percentage of TF (p = 0.001) and MMP9 (p = 0.009) -positive area from not less than 18 sections for each group (n = 6) were measured using Image Pro Plus software and shown in bar graph (right panel). The scale bar represents 100 µm. (**B**) Representative photomicrographs of aortic sinus plaque area stained with picrosirius stain for collagen (left panels). The images were captured by polarizing microscopy. The percentages of collagen positive area in total aortic sinus was measured by Image-Pro Plus software (right panel); p = 0.037.

The rupture of vulnerable plaque leads to the acute phase coronary thrombosis and causes myocardial infractions and sudden cardiac death. To understand the effect of multi-antigenic construct in stabilizing the plaque we examined the expression of known markers of plaque instability in aortic sinus. Tissue factor (TF) is a cofactor involved in activation of the coagulation pathway which can lead to the formation of thrombus. The percentage of tissue factor (TF) expression in aortic sinus as studied by immunohistochemistry, was significantly lesser in comparison with control (0.61 ± 0.03% v/s 0.93 ± 0.04%, p = 0.0001) (Fig. 3A). Matrix metallo proteinase 9 (MMP9) is one of the major reason for plaque instability as they digest the collagen in intimal layers. AHC treatment resulted in significantly lowering the levels of MMP9 in relation to the control group (0.69 ± 0.08% v/s 1.19 ± 0.110%, p = 0.0009) (Fig. 3A). In corroboration with lower MMP9 the collagen content was found to be higher (p = 0.002) in the treated sinus (67.19 ± 2.71%) when compared to control group (52.2 ± 1.86%) (Fig. 3B).

### AHC treatment decreases macrophage apoptosis

**Figure 4:**
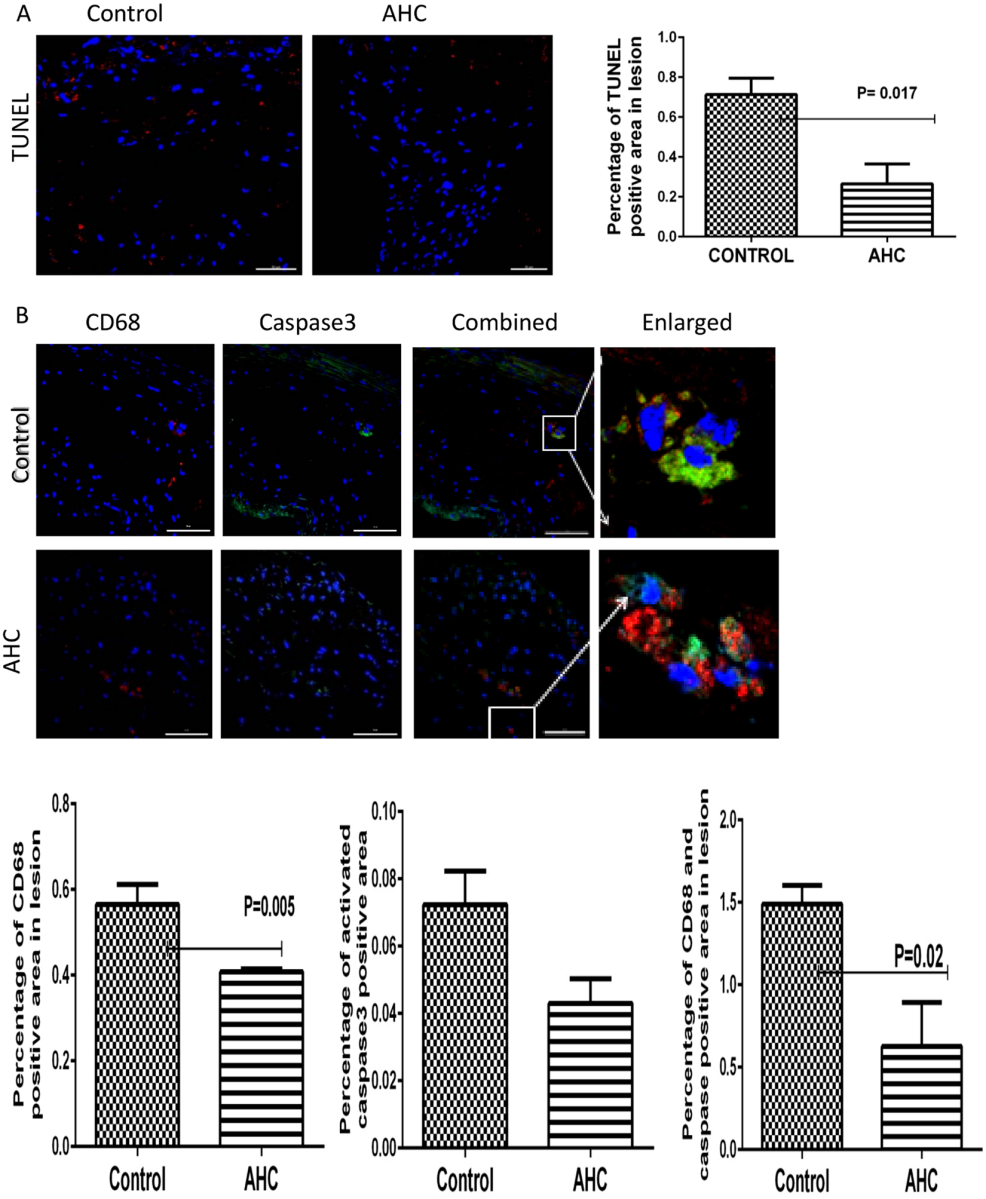
Oral administration of AHC to established disease reduces macrophage-mediated apoptosis. (**A**) Representative photomicrograph showing immunofluorescence staining of aortic sinus sections with TUNEL. Percentage of TUNEL -positive area from not less than 18 sections for each group (n = 6) were measured using Image Pro Plus software and shown in bar graph (right panel). The scale bar represents 200 µm. p = 0.017. (**B**) Representative photomicrograph showing immunofluorescence staining of aortic sinus sections with CD68 and activated caspase3 (red). Percentage of CD68 (P = 0.05), activated caspase3 and caspase3 positive CD68 co-localised area (P = 0.02) from not less than 18sections for each group (n = 6) were measured using Image Pro Plus software and expressed (right panel). The scale bar represents 200 µm.

Plaque necrosis is mainly contributed by apoptosis of macrophages and smooth muscle cells. We observed significantly lower TUNEL positive area in treated group when compared to control group (0.27 ± 0.10% v/s 0.71 ± 0.08%, p = 0.017) suggesting that the decreased acellular area observed in the plaque could be due to reduction in apoptosis (Fig. 4A). We co stained macrophages with activated caspase3 to understand the nature of the cells which are undergoing apoptosis. The overall macrophage infiltration to the plaque area was significantly lower for AHC treated group (0.40 ± 0.01% vs 0.57 ± 0.04%, p = 0.005). Macrophage apoptosis was significantly lower than the control group (0.63 ± 0.27% v/s 1.49 ± 0.11%, p = 0.02) (Fig. 4B).

### Possible mechanism of T_regs_ mediated reduction in plaque instability

**Figure 5:**
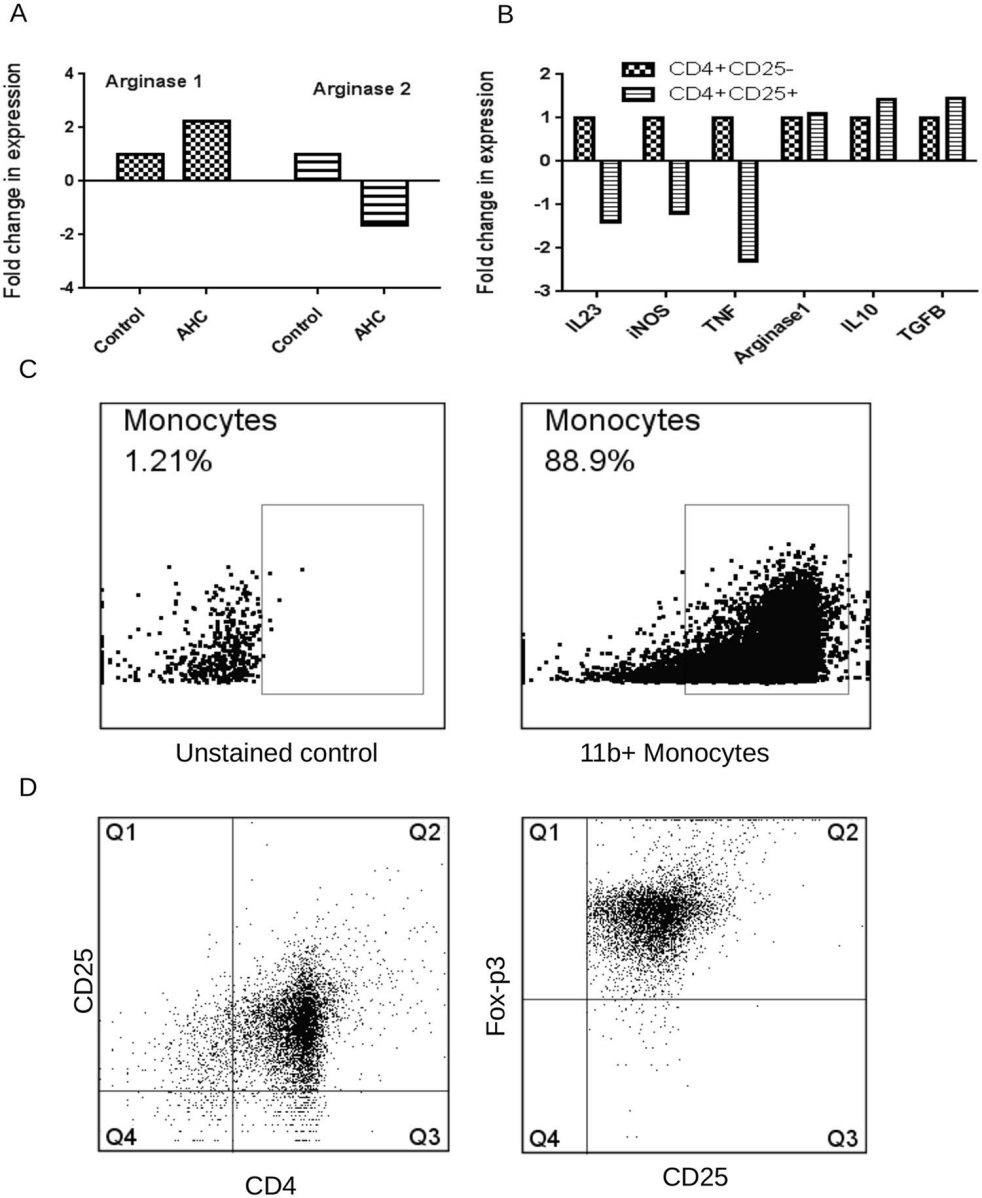
Oral administration of AHC polarizes the macrophages into M2 phenotype. (**A**) mRNA levels of Arginase1 and Arginase2 in the ascending aorta were quantified using RT-PCR analysis and normalized with GAPDH. Fold changes in expression of the AHC immunized mice relative to control mice are shown, n = 6 per group. (**B**) mRNA levels of IL-23, Inos, TNF-α, Arginase1, IL-10 and TGF-β monocytes co cultured with CD4^+^CD25^+^ Tregs and CD4^+^CD25^−^ T effector cells from AHC treated animals were quantified using RT-PCR analysis and normalized with GAPDH. Fold changes in expression of the CD4^+^CD25^+^ cultured cells relative to CD4^+^CD25^−^ are shown, n = 6 per group. (**C**) Flow cytometry dot plot of purified CD11b+ cells from healthy animals using CD11b+ microbeads (Miltenyi Biotech) (Right panel). (**D**) Flow cytometry dot plots of Treg cells (CD4+ CD25+) purified from AHC treated animals using regulatory T Cell Isolation Kit (Miltenyi Biotech). The purified cells were labeled with Foxp3 (right panel).

Our results suggest that oral administration of AHC molecule increases T_regs_ cells in the plaque which is a key regulator of inflammation. While characterizing the gene expression of different inflammatory mediators in the plaque to understand the possible mechanism of this protection, we found a 2.3 fold increase in arginase1 expression, which is known to be marker of anti-inflammatory macrophages while the relatively 1.6 folds lower expression of arginase2 which is a marker of inflammatory macrophages (Fig. 5A). These results led us to speculate if the Tregs could have a role in alternate activation of macrophages or change the expression of pro/anti inflammatory markers in the plaque. To understand the role of immune regulation of macrophages, we purified CD4^+^CD25^+^cells and CD4^+^CD25^−^ cells from AHC treated mice and co-cultured them with CD11b^+^ monocytes isolated from normal mice. We found an increase in expression of markers related to M2 macrophage phenotype in the presence of CD4^+^CD25^+^cells compared to CD4^+^CD25^−^ cells as determined by the relative higher expression levels of arginase1 (1.1 folds), IL-10 (1.43 folds) and TGF-β (1.45 folds) while lower expression of IL-23 (1.4 folds), iNOS (1.2 folds) and TNF-α (2.3 folds) which are predominantly expressed by inflammatory M1 macrophages (Fig. 5B).

## Discussion

In this study we show alteration of lesion phenotype by oral administration of a multi-antigenic molecule-AHC as a therapy for established atherosclerotic disease in a double knockout mice model (Apob^tm25gy^ Ldlr^tm1Her^). Several Studies have shown the protection against atherosclerosis by modulating the immune system using ApoB and HSP60 peptides in early stages of the disease but a curative effect of immune modulation has not been studied so far^11–18^.

We have earlier established that oral treatment with the multi-antigenic construct AHC induces immune tolerance and prevents disease development in both mice and rabbit^18,30^. In the present study, we wanted to understand if tolerance can be induced in animals who have already developed the disease and therefore examined the number and functions of Treg cells in treated and control animals in mice with established atherosclerosis. The number of Tregs in spleen as seen by CD4^+^CD25^+^FoxP3 positive cells were significantly higher in the treated animals at the end of the 22 weeks. These Tregs could prevent the proliferation of effector T cells in the presence of peptides when compared to control group. The regulatory T cells were also found to infiltrate into the aorta to mediate their anti inflammatory response. Although oral administration increased the number of Tregs in aorta as well as secondary lymphoid organs, it was not effective in reducing the growth of plaque in the aortic sinus. This is in contrast to our earlier observations on plaque reduction by oral tolerance in young animals^18^. In the present study, we allowed the animals to develop atherosclerosis for 10 weeks, which resulted in almost 25% of the sinus covered with plaque before inducing immune tolerance. Immune regulation induced by tolerance was probably not sufficient to counterbalance the overwhelming inflammation which had already set in by the time the treatment was initiated. Another important observation was a significant reduction in plaque area in the innominate or the brachiocephalic artery (BCA) in the treated animals. In mice, atherosclerotic lesions initiate first in the aortic sinus followed by BCA and then in the thoracic and abdominal aorta^31,32^. Lesions at different locations are reported to show variable sensitivity to manipulations as the lesion age is different at each site^33^. In two independent studies on the effect of liver X receptor agonist, for atherosclerosis progression, sampling after 2 months showed an anti atherosclerotic effect in aortic sinus, while it was observed only in innominate artery and not in aortic sinus after 3 months^34,35^. It is possible that lesions in the aortic sinus become too progressive to be affected by any treatment, while innominate artery lesions, which are initiated later, could be more sensitive to the manipulation, especially when sampled at late atherosclerosis^32^. We observed that the morphology of the plaque was very different in the treated animals and showed significant reduction in markers associated with plaque destabilization. Lesser number of breaks in the fibrous cap of the plaque was also indicative of stability due to AHC treatment.

The role of regulatory T cells controlling immune response in atherosclerosis has been shown by several studies in mice^36–38^. But the molecular mechanisms by which these cells mediate a protective role are still not completely understood. Some of the mechanisms by which Tregs exert their protective effect are thought to be due to suppression of inflammatory T effector cells^39^ mediated through anti inflammatory cytokines^40^ by inhibiting dendritic cell maturation^41^ and by suppressing MCP-1 expression and monocyte recruitment into plaque^42^. Recent studies have also revealed a role of Tregs in cholesterol metabolism and reduction in circulating lipid levels^42,43^.

To understand the mechanism of protection induced by AHC oral immunotherapy, we studied the macrophage polarization. Accumulation of cholesterol filled macrophages and their balance in the plaque is dynamic and that both macrophage numbers and the inflammatory phenotype influence the fate of the plaque^44^. M1 macrophages express genes, which are pro-inflammatory and cytotoxic including inducible nitric oxide synthase (iNOS), IL-12, IL-23, TNF-α, class II MHC, and the chemokines IL-8 and CCL2, participating in killing intracellular parasites and tumor development whereas anti-inflammatory M2 macrophages produce cytokines and substances involved in repairing function, typically arginase/ornithine, EGF, VEGF, and TGF-β, IL-10, IL-4 and mannose receptor^45–48^. Co culture of Tregs with monocytes was earlier shown to reduce foam cell formation^49^. We observed an alternate activation of monocytes induced by the regulatory T cells purified from AHC treated animals, suggesting that one of the anti inflammatory activities of Tregs could be mediated by the conversion of M1 to M2 macrophages. Treg and monocyte coculture from healthy individuals was earlier shown to induce a M2 phenotype in monocytes^50^.

M2 macrophages promote tissue remodeling, angiogenensis and immune regulation^51^. Deleterious effects of M1 macrophages are likely to be counterbalanced by the anti inflammatory activity of M2 macrophages as M1 mcrophages are predominantly found in vulnerable shoulder regions of the lesions^52^ whereas both M1 and M2 macrophages are seen in fibrous cap^53,54^. Metalloproteinases co localize with M1 macrophage markers in human atherosclerotic lesions while plaque regression correlates with M2 activation^55,56^ suggesting that plaque instability might be a consequence M1 and M2 imbalance^57^. Our results further support these hypothesis as we show that oral tolerance to atherogenic antigens induces antigen specific Tregs which reduce plaque inflammation by inducing a macrophage polarization to M2 phenotype, reducing matrix metalloproteases, macrophage apoptosis and reducing markers associated with plaque instability.

Our study suggests that immune modulation is an effective option to reduce inflammation in advanced atherosclerosis. Although this therapy may not drastically reduce the plaque development it can induce a more stable plaque. We had earlier reported a reduction in progression of disease in rabbits, wherein at the time of treatment 12% of sinus was covered with plaque^30^, suggesting that immune modulation may still reduce the disease progression if treated at an early stage.

These observations are very critical as in a clinical setting there will always be patients with different stages of the disease having variable percentage of plaque already deposited in their coronary artery. The tolerance bought by oral immune therapy to a established disease using AHC multi-antigenic molecule can stabilize the plaque and also can prevent the plaque rupture of disease which is a very important aspect in clinical therapy.

## Methods

### Animals

A group (6–8 animals) of C57BL/6 background mice knocked out for Apob^tm2Sgy^/Ldlr^tm1Her/J^ (Jackson laboratories) were used for all the experiments approved by the Institutional Animal Ethics Committee of the Thrombosis Research Institute (Registration Number: 1261/c/09/CPCSEA) in compliance with Government of India guidelines and conform to the Guide for the Care and Use of Laboratory Animals published by the US National Institutes of Health (NIH Publication, 8th Edition, 2011).

### Materials

Multi-antigenic construct consisting of APOB, HSP60 and chlaymydia pneumonia outer membrane protein and a diet rich in fat termed as HFD (21% anhydrous milk fat and 1.25% cholesterol).The construction and purification of AHC molecule is described in detail in the supplementary section^30^.

### Generation of multi-antigenic construct AHC

The multi antigenic construct was constructed as described earlier^29^. The epitope derived from human ApoB100 (peptide sequence: I_688_EIGLEGKGFEPTLEALFGK_707_, numbered including signal peptide) was linked at the N-terminal of dendroaspin Epitope from hHSP60 (peptide sequence: A_153_ELKKQSKPVT_163_) was used to replace the dendroaspin loop III. *Cpn* sequence derived from the major outer membrane protein (peptide sequence: G_67_DYVFDRI_74_) and polymorphic outer membrane protein 5 (peptide sequence: Q_283_AVANGGAI_291_) was linked at the C terminal of dendroaspin. The construct was cloned in pET15b expression vector and purified using anion exchange column as described in the supplementary section.

### Generation of atherosclerosis in experimental mice

Knock out animals aged for 5–6 weeks were fed on high fat diet for 10 weeks and orally treated with multi-antigenic construct AHC for five alternate doses (1 µg/dose) or ovalbumin (1 µg/dose) as control and were continued on HFD for another 10 weeks. Six animals were sacrificed after 10 weeks of HFD to serve as baseline before treatment. The design of the experiment is shown in Fig. 2A. An overdose of isoflurane inhalant anesthetic (15%) as per American Veterinary Medical Association guidelines (June 2007) was used for sacrificing the mice humanely and the organs were collected for histochemical analysis.

### Assessment of lesion area

A transversal cut approximately 4 mm away from the apex with a scalpel on the heart which is perpendicular to the ascending aorta was made (approx 45° Inter ventricular septum angle). For lesion analysis not less than 6 sections 80 µm apart were stained with Elastica van Geison (EVG) were considered for quantification to avoid false positive lesion area. From each animal 25–30 sections could be collected and each slide was numbered for convenience. The same slide numbers were taken from each experimental group for quantification. The total area of sinus, area covered by lesion and the percentage lesion area were quantified for each section. The average values for each mice was calculated from not less than 6 section. Results for were represented as the average of all the animals in the experimental group. Hematoxylin and eosin staining was used to quantify the acellular area as necrotic core in the aortic sinus. Image-Pro Plus software (Media Cybernetics, Bethesda, USA) was used for morphometric analysis as described earlier^18^.

### Analysis of aortic sinus by Immunohistochemistry

Immunofluorescence was used to quantify specific antigens and cytokines in the aortic sinus. The frozen sections were taken in slides coated with 5% amino-silane and permeabilized using 0.2% of triton X 100 for 30 min, fixed with ice-cold acetone, blocked with 5% serum incubated with primary antibodies followed by appropriate secondary antibody (Alexa-633 tagged Invitrogen), Vector Shield mounted sections were imaged using a Leica DMI 4000 B confocal microscope and the analysis was done using Image-Pro software (Media Cybernetics, Bethesda, USA). The antibodies used were specific for tissue factor (TF), matrix metalloproteinase 9 (MMP9), TUNEL, CD68, activated caspase3, CD4 and Foxp3 from mice. Macrophage apoptosis was quantified by co localization of CD68 and Caspase 3 while Tregs were enumerated by CD4 and FOxp3 co staining.

### Picrosirius staining for collagen content

Collagen content within the plaque was measured for treated and control group by taking at least six sections per animal. The de-waxed and hydrated sections followed by staining with Weighert’s haematoxylin for 8–10 minutes were washed for 10 minutes in running tap water and stained with picrosirius red for one hour. The slides were washed, rinsed with acidified water and dehydrated in 2–3 changes of 100% ethanol quickly, cleared in xylene and mounted with DPX. Images were captured using a polarizing microscope (Olympus BX-51). Quantification was performed Image-Pro Plus software (Media Cybernetics, Bethesda, MD).

### Flow Cytometry

Flow cytometry analyses of spleen cells were performed by FACS Canto II using FACS DIVA software (Becton–Dickinson, New Jersey, USA) and FLOWJO software (Tree star Ltd, Oregon, USA). The antibodies used were as follows: Fluorescein isothiocyanate (FITC)-conjugated CD4 (clone RM4-5), allophycocyanin (APC)-anti CD25 (clone PC61.5), PE- IFN-γ (clone XMG1.2) all from e-Biosciences San Diego, CA, USA. APC-Cy™ 7 IL-17A (clone TC11-18H10, BD pharmingen, San Jose, USA), phycoerythrin (PE)-anti-fork head box p3 (Foxp3) (clone NRRF-30). Surface and Intracellular staining were performed according to standard procedures at a density of 10^5^ cells/100 µL according to the manufacturer’s instructions.

### Gene expression analysis of aortic cytokines by RT-PCR

The total RNA from the ascending part of the aorta using TRIzol® Reagent from 6 control and 6 treated mice was isolated and mRNA was reverse transcribed using invitrogen CDNA synthesis kit and polymerase chain reaction (RT-PCR) was performed using Superscript® RT-PCR kit (Invitrogen, Carlsbad, USA) using an ABI PRISM 7900HT fast real time PCR system (Applied Biosystems, California, USA). The list of primers used for the study was listed in Table 1.

**Table 1:** The list of primers used in the study.

IL23-F	CCC GGG TTA GGC TGC CTG GCG GAC A
IL23-R	GAG TCA AGC TGT TGG CAC TAA GGG CTC
INOS-F	CAG CTG GGC TGT ACA AAC CTT
INOS-R	CAT TGG AAG TGA AGC GGT TCG
TGF-β1-F	TTG CTT CAG CTC CAC AGA GA
TGF-β1-R	TGG TTG TAG AGG GCA AGG AC
IL10-F	GTG GCA GTG AAG ACC ATG AAG TTG
IL10-R	GAA CTC CGG GAT AGG GAG TCA T
GAPDH-F	AAC TTT GGC ATT GTG GAA GG
GAPDH-R	ACA CAT TGG GGG TAG GAA CA
TNF-α-F	ATG AGC ACA GAA AGC ATG ATC
TNF-α-R	TAC AGG CTT GTC ACT CGA ATT
FoxP3-F	CCC ATC CCC AGG AGT CTT G
FoxP3-R	ACC ATG ACT AGG GGC ACT GTA
CTLA-4-F	GCT TCC TAG ATT ACC CCT TCT GC
CTLA-4-R	CGG GCA TGG TTC TGGBATC A
Arginase1-F	GGA ACC CAG AGA GAG CAT GA
Arginase1-R	TTT TTC CAG CAG ACC AGC TT
Arginase2-F	ACC AGG AAC TGG CTG AAG TG
Arginase2-R	TGA GCA TCA ACC CAG ATG AC

### Functional immunoassays and Proliferation assays

Peptide specific effector T-cells, were generated and used as mentioned earlier^30^. The effector cells were labeled with 10 μM 5,6-carboxyfluorescein diacetate succinimidyl ester (CFSE) (Sigma chemicals, St. Louis, USA). The spleen cells were isolated from both AHC treated and control mice and non adherent lymphocytes were used to discriminate the effector and regulatory population using regulatory T Cell Isolation Kit in auto MACS pro (Miltenyi Biotech). Effector T cells (1 × 10^5^) and regulatory cells were taken in equal ratio and cultured in 96 well plates in the presence of 10 μg/mL of peptides in X-vivo 20 serum free medium (Lonza, Basel, Switzerland). After 5 days of incubation, cells were stained with CD4-APC (eBiosciences, California, USA)^20^. CD4 effector cells proliferation was measured by CFSE dilution using flow cytometry. Spleen cell proliferation assay was carried out using Roche cell proliferation BrdU assay kit as described earlier^18^. Spleen cells from the both control and treated group were cultured for 54 h and labeled with 10 µl BrdU reagent for the another 18 h of culture. The cells were fixed and the DNA denatured by adding FixDenat fixing and denaturation solution, followed by anti-BrdU-POD antibody which binds to the BrdU incorporated into the newly synthesized cellular DNA. The immune complexes formed by the tetra methyl benzidine (TMB) substrate reaction were dispersed and the absorbance was recorded by an ELISA reader.

### Monocyte differentiation to anti-inflammatory M2 macrophages

The Tregs were isolated from spleens of animals treated with AHC. CD4^+^CD25^+^ and CD4^+^CD25^−^ cells were purified using regulatory T Cell Isolation Kit in auto MACS pro (Miltenyi Biotech, Cologne, Germany). Mononuclear cells were isolated from the spleen of untreated healthy mice. We used healthy male mice since the treatment might have some influence on the monocytes. Mononuclear cells from the spleen were pooled from three individual mice and purified using CD11b micro beads in auto MACS pro (Miltenyi Biotech, Cologne, Germany). Purified 11b positive cells (1 × 10^6^ cells) were co-cultured with equal numbers of either CD4^+^CD25^−^ or CD4^+^CD25^+^ cells from AHC treated animals for 7 days. Loosely adherent T cells were removed from the culture and the adherent macrophages were washed twice with PBS. Total RNA from the macrophages was extracted using TRIzol® and the relative gene expression of M1 macrophage marker (IL-23, inducible nitric oxide synthase (iNOS) and TNF-α) and M2 macrophage marker (arginase1, TGF-β and IL-10) were studied using QRTPCR.

### Statistical Analysis of Data

Graph Pad prism software version 5.01(GraphPad Software, Inc., La Jolla, CA, USA) was employed for statistical analysis and results were expressed as Mean ± SEM. The final data represents the average of 6 animals. For each animal multiple sections were quantified to get the mean value. For the quantification of lesion area, not less than 6 sections per animal were taken for the analysis. The mean area of lesion was calculated per mice and the results are represented as the average of 6 mice per group. For immunohistochemical analysis, 3–5 sections were taken per mice and the results were computed as average of 6 mice per group. Flow cytometry and other *in vitro* experiments were performed from cells isolated from individual mice and results represented as average of 6 mice/group. Mann-Whitney test with statistical significance at *P* < 0.05 were considered for showing the differences between control and treated groups.

## Electronic supplementary material

Supplementary Information
