# Author Correction: Immune regulation by oral tolerance induces alternate activation of macrophages and reduces markers of plaque destabilization in Apob^tm2Sgy^/Ldlr^tm1Her/J^ mice

**DOI:** 10.1038/s41598-018-34060-z

**Published:** 2018-10-24

**Authors:** Lakshmi Narasimha Thota, Thiruvelselvan Ponnusamy, Sheena Philip, Xinjie Lu, Lakshmi Mundkur

**Affiliations:** 1 Research Scholar, Manipal University, (Madhav Nagar, Manipal) at Molecular Immunology Unit Thrombosis Research Institute, Bangalore, India; 2 Molecular Immunology unit, Thrombosis Research Institute, Bangalore, India; 3 Molecular Immunology unit, Thrombosis Research Institute, London, UK

Correction to: *Scientific Reports* 10.1038/s41598-017-04183-w, published online 21 June 2017

In the original version of this Article, Lakshmi Narasimha Thota and Thiruvelselvan Ponnusamy were incorrectly affiliated with ‘Molecular Immunology unit, Thrombosis Research Institute, Bangalore, India’. The correct affiliation for Lakshmi Narasimha Thota and Thiruvelselvan Ponnusamy is listed below:

Research Scholar, Manipal University, (Madhav Nagar, Manipal) at Molecular Immunology Unit Thrombosis Research Institute, Bangalore, India.

This has now been corrected in the HTML and PDF versions of this Article, and in the accompanying Supplemental Material.

