# Supplementary material for: Immune regulation by oral tolerance induces alternate activation of macrophages and reduces markers of plaque destabilization in Apobtm2Sgy/Ldlrtm1Her/J mice
Source: Sci Rep. 2017 Jun 21;7:3997. doi: 10.1038/s41598-017-04183-w (PMC5479867; doi:10.1038/s41598-017-04183-w)
Supplement: Supplementary file 1 — Supplementary Information [file 41598_2017_4183_MOESM1_ESM.pdf]

Immune regulation by oral tolerance induces alternate activation of macrophages and reduces markers of plaque destabilization in $Apob^{tm2Sgy}/Ldlr^{tm1Her/J}$ mice

Lakshmi Narasimha Thota[1], Thiruvelselvan Ponnusamy[1], Sheena Philip[2], Xinjie Lu[3], Lakshmi Mundkur*[2]

[1] Research Scholar, Manipal University, (Madhav Nagar, Manipal) at Molecular Immunology Unit Thrombosis Research Institute, Bangalore,India.

[2] Molecular Immunology unit, Thrombosis Research Institute, Bangalore, India.

[3] Molecular Immunology unit, Thrombosis Research Institute, London UK

***Corresponding author:**

Dr. Lakshmi A Mundkur, Thrombosis Research Institute (Bangalore), Narayana Hrudayalaya, 258/A, Bommasandra Industrial Area, Anekal Taluk, Bangalore-560099, India, Ph:080-27835303; Fax- 080-27835302

E-mail: lakshmi.mundkur@triindia.org.in, laksmundkur@gmail.com

Methods

Generation of recombinant construct AHC

The detailed structural dendroaspin carrier for incorporating multi antigens was described earlier[1]. The epitope derived from human ApoB100 (peptide sequence: I_{688}EIGLEGKGFEPTLEALFGK_{707}, numbered including signal peptide) was incorporated into the N-terminal of dendroaspin with a poly-glycine linker between ApoB peptide and dendroaspin. Epitope from hHSP60 (peptide sequence: A_{153}ELKKQSKPVT_{163}) was incorporated in dendroaspin loop III as a replacement of wild-type loop III sequence. *Cpn* sequence in a combination form derived from the major outer membrane protein (MOMP) of the *Cpn* (peptide sequence: G_{67}DYVFDRI_{74}) and polymorphic outer membrane protein (Omp) 5 of *Cpn* (peptide sequence: Q_{283}AVANGGAI_{291}) at the C terminal of dendroaspin.

Cloning of untagged AHC gene insert and transformation

The genes were synthesized by Genescript, USA Inc under confidential agreement and cloned into a PUC57 vector. The genes of constructs AHC was amplified by PCR and cloned in a pGEX-3X vector as a GST tagged multi antigenic construct as described earlier[2]. The AHC gene was sub cloned in pET15b expression vector by amplifying the 387 bp insert using forward primer 5'-GAGCCATGGTAGGGATCCATATCGAAGG-3' and reverse primer 5'-GTGCTCGAGTCATCACGAGAATTCTC-3' from the pGEX vector at NcoI and XhoI sites. The protein was expressed in Rosetta cells and induced using IPTG. The recombinant protein was purified from 2 liter fermenter culture

Purification of Protein

Inclusion bodies solubilization and dilution

Inclusion bodies were washed twice with 20 mM Tris – HCl,1mM EDTA, pH 8.0 0.1 Triton X-100 to remove cellular proteins and solubilized in 6 M GuCl at 2 to 8 degree Celsius at the concentration of 1g wet weight/50ml (~ 20 mg/ml) for 3 to 4 hrs. After dissolution it was centrifuged at 12000 rcf to get clear supernatant. The supernatant was slowly diluted in 20 mM Tris – HCl pH – 8.0 containing 10 mM glutathione oxidized and 4 mM glutathione reduced buffer (the final dilution was 2mg/ml) for at least 2 hrs at 2 to 8 degree Celsius. We developed a two-step chromatography purification process, using gel filtration chromatography (Sephadex G100) followed by anion exchange chromatography (Q Sepharose FF). The reason for using two chromatography steps was to first refold the protein followed by purification.

Chromatography

Desalting

As GuCl interferes with anion exchange chromatography and reduces the resolution, binding capacity we performed desalting using HiPrep (53 ml column volume) desalting column run at 100 cm/hr with sample load of 12 % (6.5 ml per desalting run). The column was equilibrated with 50 mM Tris – HCl, pH 8.0 with 3 column volumes. The desalted protein solution was pooled from all the runs and used for next step.

Anion Exchange chromatography (Q Sepharose FF)

QFF was equilibrated at 15o cm/hr (2.5 ml/min) with50 mM Tris – HCl, pH 8.0 (buffer A) with five column volumes. The desalted protein sample was loaded at 125 cm/hr (in buffer A). The elution was performed using a linear gradient over 25 column volume using 50 mM Tris-HCl, pH 8.0 + 1 M NaCl (buffer B)**.** The protein of interest eluted in 25 CV linear gradients and concentrated and aliquoted before storing at -80°c. The total yield from the 2 litre culture was 5mg.

Multiantigenic (AHC) protein usage

The protein concentration was determined using Bradford method each time before using for animal experiments to make sure the intake concentration is the same throughout the experiment.

Atherosclerotic Lesion Assessment

Quantification of atherosclerotic lesions was carried out as per the protocol approved by the Animal Models of Diabetic Complications Consortium (http://www.diacomp.org). An overdose of isoflurane inhalant anesthetic (15%) as per American Veterinary Medical Association guidelines (June 2007) was used for sacrificing the mice humanely and the organs were collected for histochemical analysis. Hearts were perfused with 10 mL of PBS and collected in either optimal cutting temperature (OCT) medium (Tissue Tek, Leica, Germany) or buffered phenol. A cryotome (Leica CM 1900 UV Cryotome) was used to cut 10 µm sections from aortic root under frozen conditions, while 5uM sections were cut from hearts in neutral buffered phenol (NBF) after embedding in paraffin blocks. Sections were collected in coated slides when the sinus appeared as three bipartite valve bases with attached leaflets. Two to three sections were collected in a single glass slide. Sections were collected till the atherosclerotic lesions were no longer visible. In all around 25-30 sections could be collected from each mice and each slide was numbered for convenience. For lesion analysis not less than 6 sections 80 µm apart were stained with Elastica van Geison (EVG). The same slide numbers were taken from each

experimental group for quantification. The total area of sinus, area covered by lesion and the percentage lesion area were quantified for each section using Image-Pro Plus software (Media Cybernetics, Bethesda, MD). The average values for each mice was calculated from not less than 6 section. Results for were represented as the average of all the animals in the experimental group[3]. Plaque necrosis was quantified by measuring the size of the hematoxylin and eosin-negative acellular area, as described previously[4].

Results:

Effect on disease progression upon oral administration of AHC in established disease

Figure 1

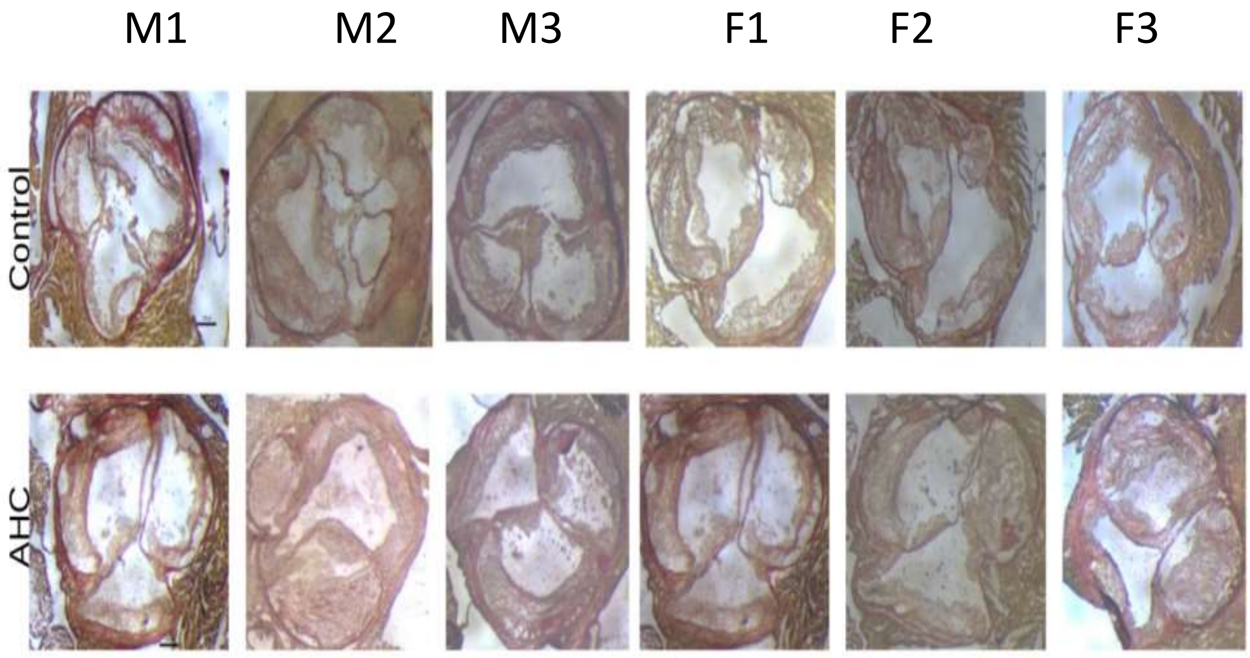

Representative photomicrographs of aortic sinus plaque area stained with Elastica van Gieson (EVG) stain from 6 mice. The hearts are sectioned at the end of the study. The percentages of plaque area in total aortic sinus and necrotic core area were measured and quantified. M1-3:Male, F1-3 : Female

Table 1: Quantification of percentage lesion area from individual mice

	HFD GROUP	AHC GROUP
M1	62.21	50.22
M2	66.01	59.81
M3	55.14	56.41
F1	52.68	64.35
F2	68.73	63.70
F3	60.01	50.24
Group average± SEM	**60.8 ± 2.5**	**57.4 ± 2.56**

Quantification of lesion area from control and treated groups. Each value represents the average of not less than 6 sections from each animal. Group average was computed from the average of 6 animals

References

1 Sutcliffe, M. J., Jaseja, M., Hyde, E. I., Lu, X. & Williams, J. A. Three-dimensional structure of the RGD-containing neurotoxin homologue dendroaspin. *Nature Structural & Molecular Biology* **1**, 802-807 (1994).

2 Lu, X. *et al.* Impact of multiple antigenic epitopes from ApoB100, hHSP60 and Chlamydophila pneumoniae on atherosclerotic lesion development in Apob(tm2Sgy)Ldlr(tm1Her)J mice. *Atherosclerosis*, doi:10.1016/j.atherosclerosis.2012.07.021 (2012).

3 Venegas-Pino, D. E., Banko, N., Khan, M. I., Shi, Y. & Werstuck, G. H. Quantitative analysis and characterization of atherosclerotic lesions in the murine aortic sinus. *J Vis Exp*, 50933, doi:10.3791/50933 (2013).

4 Feng, B. *et al.* Niemann-Pick C heterozygosity confers resistance to lesional necrosis and macrophage apoptosis in murine atherosclerosis. *Proc Natl Acad Sci U S A* **100**, 10423-10428, doi:10.1073/pnas.1732494100 (2003).
